# Annexin A2 Coordinates STAT3 to Regulate the Invasion and Migration of Colorectal Cancer Cells *In Vitro*

**DOI:** 10.1155/2016/3521453

**Published:** 2016-05-04

**Authors:** Dianhui Xiu, Lin Liu, Fengli Qiao, Haishan Yang, Lu Cui, Guifeng Liu

**Affiliations:** ^1^Department of Radiology, China-Japan Union Hospital, Jilin University, Changchun 130033, China; ^2^Department of Ultrasonography, China-Japan Union Hospital, Jilin University, Changchun 130033, China; ^3^China-Japan Union Hospital, Jilin University, Changchun 130033, China

## Abstract

The present study aimed to reveal the expression of STAT3 and Anxa 2 in CRC specimens and to investigate the effects of STAT3 and Anxa 2 signaling on the proliferation, invasion, and migration in CRC Caco-2 cells. Results demonstrated that both Anxa 2 and STAT3 were highly expressed in CRC specimens in both mRNA and protein levels, with or without phosphorylation (Tyrosine 23 in Anxa 2 and Tyrosine 705 in STAT3). And the upregulated Anxa 2 promoted the phosphorylation of STAT3 (Tyrosine 705) in CRC Caco-2 cells. The upregulated Anxa 2 promoted the proliferation, migration, and invasion of Caco-2 cells in vitro. Moreover, the STAT3 knockdown also repressed the proliferation, migration, and invasion of Caco-2 cells. In conclusion, the overexpressed Annexin A2 regulated the proliferation, invasion, and migration in CRC cells in an association with STAT3.

## 1. Introduction

Colorectal cancer (CRC) is one of the most commonly lethal cancers and is considered as the third diagnosed cancer. It causes about 1.2 million new cases and 655,000 deaths every year worldwide [1, 2]. Studies on CRC pathogenesis have been focused on* in situ* inflammatory microenvironment. The pro- or antitumor effect of immunologic factors has been indicated to play key roles in the progression of cancers, including CRC [3]. Determinants that link immune system with tumor cells make the oncogenic factors activating, while inactivating tumor suppressors. Particularly, Signal Transducer and Activator of Transcription 3 (STAT3) is activated during the tumorigenesis of CRC [4]. STAT3 consists of N-terminal domain, coiled-coil domain, DNA-binding domain, and Src homology 2 and transactivation domain [5, 6] and plays versatile roles in cell survival, apoptosis, migration, and differentiation [6]. And STAT3 takes intrinsic activator effects [7, 8] in cancer inflammation and in the regulation of the tumor microenvironment.

Annexin A2 (also known as p36, Anxa 2, or annexin II) is a 36 kDa calcium-dependent phospholipid-binding protein [9] and has been well characterized as a receptor for plasminogen activator [9–11]. Anxa 2 is indicated to be involved in endocytosis [12, 13], membrane trafficking [14] in normal cells, and the proliferation, adhesion, migration, invasion, or angiogenesis of cancer cells [15, 16]. The Anxa 2 overexpression has been observed in many types of tumor tissues and has been found to contribute to cancer progression [15, 16]. It was shown that the upregulated Anxa 2 promoted the invasion of breast cancer cells [17, 18] and facilitated the growth of prostate cancer [19], whereas the Anxa 2 knockdown upregulates p53 and caused cell cycle arrest of non-small-cell lung carcinoma cells [20]. Moreover, the expression of Anxa 2 had been indicated to be deregulated in CRC tissues [21, 22].

Anxa 2 is recently recognized to act as the downstreamer of EGFR signaling in breast cancer cells [23, 24]. The blocked Anxa 2 or the Anxa 2 knockdown inhibits the activation of the EGFR downstream pathways and reduces breast cancer cell migration [25]. Moreover, STAT3 is a well-known key pathway downstreamer of EGFR and a promoter to the epithelial to mesenchymal transition (EMT) in breast cancer [26], through which Anxa 2 promotes EMT in dependence on STAT3 pathway [27]. And the tyrosine 23 phosphorylation of Anxa 2 promotes the proliferation and invasion of human breast cancer cells, also via upregulating the STAT3 phosphorylation [28]. However, it is not clear whether there is a novel mechanism through which Anxa 2 interacts with STAT3 and promotes the CRC tumorigenesis.

In this study, we focused on the expression of STAT3 and on the level of Anxa 2 with or without phosphorylation in CRC and then investigated the interactions of these molecular markers in CRC cells. We also monitored the effects on the interaction of STAT3 and Anxa 2 and the effects of these molecular markers on invasion and migration in CRC cancer cells. All our findings demonstrated STAT3 coordinated Anxa 2 signaling to regulate the invasion and migration in CRC cells. It is the first report on the positive regulatory association of STAT3 and Anxa 2 signaling in CRC.

## 2. Materials and Methods

### 2.1. Human CRC Tissues

Human beings study has been approved by Ethics Committee of China-Japan Union Hospital, Jilin University. 35 CRC tissues and the paratumor tissues (2 cm away from the edge of CRC tissues) were obtained from CRC patients, with complete clinicopathologic data of these CRC patients recorded, after undergoing endoscopy and surgical resection. Detailed clinicopathologic information was listed in Table 1. And human CRC specimens in our study were allowed by patients for scientific research with written informed files and were approved by the institutional ethics committee of our hospital.

### 2.2. Cells and Cell Treatments

The human colorectal cancer cells (Caco-2) were purchased from Type Culture Collection of the Chinese Academy of Sciences (Beijing, China) and were cultured in Eagle's Minimum Essential Medium (EMEM) (Invitrogen, Carlsbad, CA, USA) containing 10% fetal bovine serum (FBS) (Invitrogen, Carlsbad, CA, USA), 100 units/mL penicillin, and 100 mg/mL streptomycin (CSPC Pharmaceutical Group Limited, Shijiazhuang, China). Caco-2 cells were cultured in a humidified incubator containing 5% CO_2_ at 37°C. pRC/CMV-Annexin A2-FLAG (Annexin A2 (+)) and pcRC/CMV (control (+)) expression vectors were purchased from Promega (Promega, Madison, WI, USA). Cell Line Nucleofector Kit T (Lonza Walkersville Inc., Walkersville, MD) was conducted to transfect plasmids into Caco-2 cells. Annexin A2 knockdown and STAT3 knockdown were conducted by using siRNAi-Annexin A2 (5′-CAAGCCCCTGTATTTTGCTGAT-3′) (for Annexin A2 silence) and scramble RNA (as control siRNA, 5′-UUCUCAGAACGUGUGACGU-3′) and shRNA STAT3/Puro (SIGMA-Aldrich) (for STAT3 knockdown, targeting sequence: 5′-GCGTCCAGTTCACTACTAAAG-3′) lentiviral vectors, respectively, with shRNAi MISSION Non-Target shRNA Control/Puro (SIGMA Aldrich, St. Louis, MO, USA) performed as negative control of gene knockdown.

### 2.3. RNA Extraction and Quantitative Real-Time Polymerase Chain Reaction (qRT-PCR)

Total RNA of tissue samples was extracted by using the RecoverAll*™* Total Nucleic Acid Isolation Kit (Ambion, Austin, USA). RNeasy kits (Qiagen, Valencia, CA) were utilized to extract total mRNA from cells. qRT-PCR was performed on ABI 7500 with SYBR Premix Ex Taq*™* Kit (Takara, Japan). GAPDH was denoted as the internal control. Data were normalized by using 2^−ΔΔCt^ method as relative quantification. The used primers were as follows: Forward primer for Annexin A2: 5′-GCTGGAGTGAAGAGGAAAGG-3′, reverse primer for Annexin A2: 5′-CACGCTCCGCTCGGTCATGA-3′; forward primer for STAT3: 5′-CATGTGAGGAGCTGAGAACG-3′, reverse primer for STAT3: 5′-TGCAGGTAGGCGCCTCAGTC-3′; forward primer for *β*-actin: 5′-GTACCCTGGCATTGCCGACA-3′, reverse primer for *β*-actin: 5′-GGACTCGTCATACTCCTGC-3′.

### 2.4. Western Blotting Assay

Protein samples were extracted from Caco-2 cells with Cell Lysis Buffer (Cell Signaling Technology Inc., Danvers, MA, USA). Then, the protein samples were subjected to 10% SDS-PAGE electrophoresis and were transferred onto nitrocellulose membranes (Millipore, Bedford, MA, USA). Western blotting was performed by using goat polyclone antibody against human Annexin A2 (Thermo Scientific, Rockford, IL, USA), or rabbit polyclone antibody against human Annexin A2 with phosphorylation (Tyrosine 23) (R&D Systems China, Shanghai, China) or rabbit polyclone antibody against human STAT3 without phosphorylation (Abcam, Cambridge, UK), against STAT3 with phosphorylated Tyrosine 705 (Abcam, Cambridge, UK) or *β*-actin antibody (Sigma-Aldrich, St. Louis, MO, USA). Horseradish peroxidase-linked mouse anti-human IgG or mouse anti-rabbit IgG (Jackson ImmunoResearch, West Grove, PA, USA) and the Super Signal West Femto (Pierce, Rockford, IL, USA) were utilized to detect the specific antigen-antibody binding.

### 2.5. Coimmunoprecipitation Assays

For the Coimmunoprecipitation assay, Caco-2 cells were lyzed with Triton X-100 (SIGMA Aldrich, St. Louis, MO, USA). The supernatants from cell lysates were incubated with the rabbit polyclone antibody against human Annexin A2 with Tyr 23 phosphorylation (Anti-p-Anxa 2) or with the rabbit polyclone antibody against STAT3 with Tyr 705 phosphorylation (Anti-p-STAT3) at 4°C overnight and then were incubated with protein A-sepharose beads (Amersham Bioscience) for 2 h at 4°C and were washed extensively. To elute the bound proteins, 100 mM glycine-HCl (pH 2.5, precipitated with 5% trichloroacetic acid) was added, and the samples were washed with ice-cold acetone and resuspended in SDS sample buffer for SDS-PAGE electrophoresis and western blotting assay.

### 2.6. Cell Proliferation and Colony Formation Assay

To assay the influence of Annexin A2 or STAT3 on the Caco-2 cell proliferation, 85% confluent Caco-2 cells (1000 cells/mL) post the manipulation of Annexin A2 or STAT3 were incubated with the EMEM containing 10% FBS for 24, 48, or 72 hours. Then cells in each group were counted. For the colony formation assay of Caco-2 cells, 4 × 10^2^ Caco-2 cells were incubated in 12-well plates and then were treated for the Annexin A2 upregulation or knockdown, or for the STAT3 knockdown. After an inoculation of 48 hours, the cell colony was stained with crystal violet (0.005%) for 20 minutes and we recorded the colony numbers by imaging J software.

### 2.7. Migration and Invasion Assay

Cell migration was assayed by transwell migration assays. Briefly, cells were grown to 1 × 10^4^ cells in Transwell migration chambers which contain upper chamber with noncoated membrane (8 *μ*m pore size, Millipore, Switzerland) and lower chamber contained media with 20% FBS as a chemoattractant. Cells in upper chamber were discarded. After 24-hour culture, migratory cells were detected by microscope in lower chamber. Invasion assay was the same as the transwell migration assays, expect for 2 × 10^4^ cells on the upper chamber coated with Matrigel (Sigma-Aldrich, St. Louis, MO, USA).

### 2.8. Statistical Analysis

The data are normalized as mean ± standard deviation. Student's *t*-test was performed to compare differences in two groups. Comparisons among multiple samples were made by ANOVA. Statistical analyses were conducted by SPSS 17.0 software. Statistically significant difference was considered when *p* < 0.05 or less.

## 3. Results

### 3.1. Overexpression of Anxa 2 and STAT3 in Colorectal Cancers

**Figure 1:**
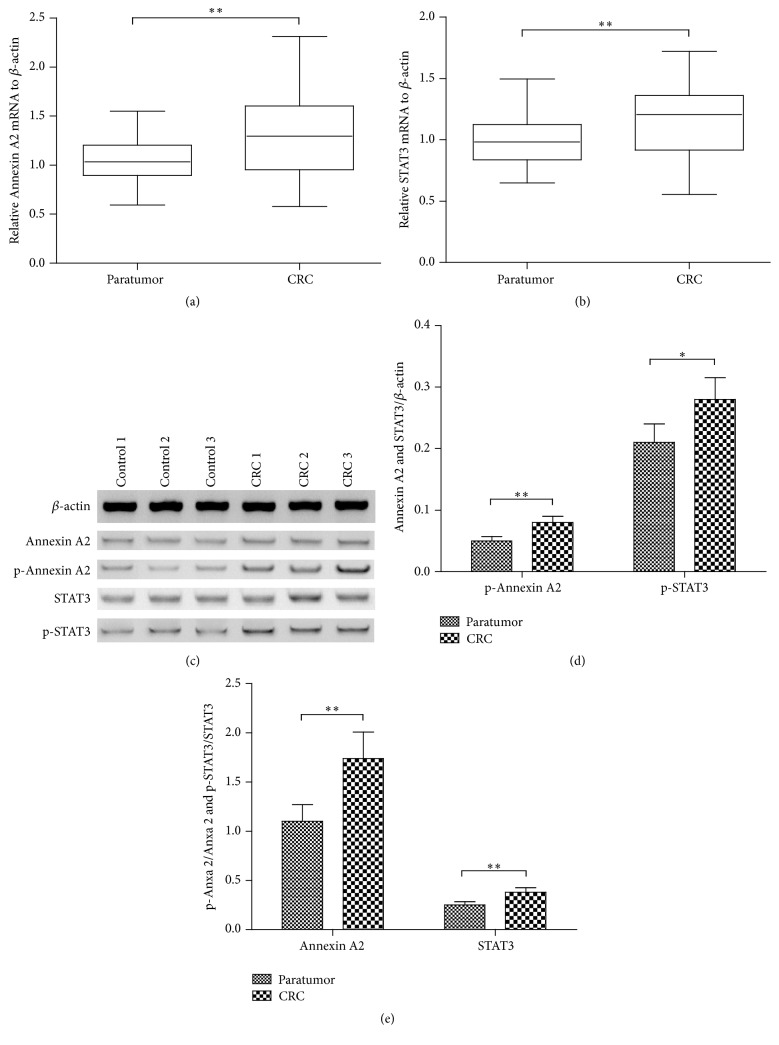
Comparisons of the expression and the phosphorylation of Annexin A2 and STAT3 between the colorectal cancer (CRC) tissue and the paratumor tissue. (a) and (b) The expression of Annexin A2 (a) and STAT3 (b) in mRNA level was assayed in CRC tissues (*n* = 35) and in paratumor tissues (*n* = 35) by relative qRT-PCR analysis; (c) representative western blot analysis of the expression in protein level and the phosphorylation of Annexin A2 (Tyrosine 23) and STAT3 (Tyrosine 705) in CRC tissues (*n* = 15) and in paratumor tissues (*n* = 15); (d) relative level of Annexin A2 and STAT3 to *β*-actin in protein level; (e) relative Annexin A2 and STAT3 with phosphorylation to nonphosphorylated Annexin A2 and STAT3. *∗* and *∗∗* represented *p* < 0.05 and *p* < 0.01, respectively.

Previous study has indicated the regulatory role of Anxa 2 [21, 22] and STAT3 [29] in CRC. However, little is known about the interaction between the two markers in the tumorigenesis of CRC. In the present study, we firstly investigated the expression of Anxa 2 and STAT3 in CRC and paratumor tissues. It was indicated in Figure 1(a) that the mRNA level of Anxa 2 was significantly higher in the CRC tissues (*n* = 35) than in the paratumor tissues (*p* < 0.01) (*n* = 35). And the STAT3 in mRNA was also markedly upregulated in the CRC tissues (*n* = 35) (*p* < 0.01, Figure 1(b)). Moreover, the western blot analysis (Figure 1(c)) indicated that both markers in protein levels were also significantly higher in the CRC tissues (*n* = 15) than in the paratumor tissues (*p* < 0.01) (*n* = 15) (*p* < 0.01 for Anxa 2 and *p* < 0.05 for STAT3, Figure 1(d)). In addition, we examined the Anxa 2 and STAT3 with phosphorylation in both groups of specimens by western blotting assay.  Figure 1(e) demonstrated that, compared to the nonphosphorylated form of Anxa 2 and STAT3, both markers with phosphorylation were also significantly higher in the CRC specimens (*p* < 0.01, resp.). All results in this study revealed the overexpression of Anxa 2 and STAT3 in CRC tissues.

### 3.2. Annexin A2 Upregulates STAT3 Phosphorylation in CRC Caco-2 Cells

**Figure 2:**
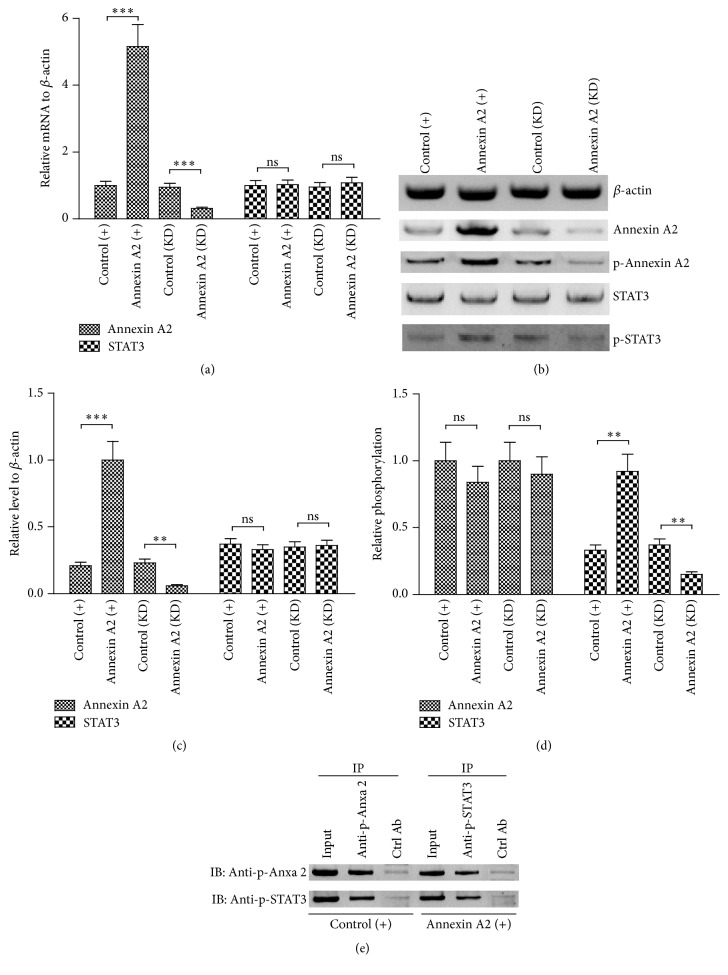
Annexin A2 positively modulated the STAT3 phosphorylation in Caco-2 cells. (a) Relative mRNA level of Annexin A2 and STAT3 in the Caco-2 cells, which were transfected with pRC/CMV-Annexin A2-FLAG (Annexin A2 (+)), pcRC/CMV (Control (+)), siRNAi-Annexin A2 (Annexin A2 (KD)), or control siRNA (control (KD)) for 24 hours; (b) western blotting assay of Annexin A2 with or without Tyrosine 23 phosphorylation and STAT3 with or without Tyrosine 705 phosphorylation in the Caco-2 cell groups of Annexin A2 (+), control (+), Annexin A2 (KD), and control (KD); (c) relative protein levels of Annexin A2 and STAT3 without phosphorylation to *β*-actin in the abovementioned groups; (d) levels of phosphorylated forms to non-phosphorylated forms of Annexin A2 and STAT3 in the abovementioned groups; (e) coimmunoprecipitation analysis of the interaction between the phosphorylated Annexin A2 (Tyr 23) and the phosphorylated STAT3 (Tyr 705). Data was averaged for triple independent experiments, *∗∗* represented *p* < 0.01, *∗∗∗* represented *p* < 0.001, and ns represented no significance.

In order to reveal if dysregulation of STAT3 was associated with Anxa 2 in CRC cells, the regulation of STAT3 by Anxa 2 was explored using gain-of-function and loss-of-function approaches. Firstly, the Anxa 2 was overexpressed or knocked down in Caco-2 cells. As shown in Figure 1(a), the mRNA of Anxa 2 was dramatically upregulated in the Caco-2 cells, which were transfected with pRC/CMV-Annexin A2-FLAG (Annexin A2 (+)), compared to the pcRC/CMV-transfected Caco-2 cells (control (+)) (*p* < 0.001). However, the Anxa 2 mRNA was markedly downregulated by the siRNAi-Annexin A2 transfection (Annexin A2 (KD)), in comparison with the control siRNA-transfected Caco-2 cells (control (KD)) (*p* < 0.001). And such upregulation in Annexin A2 (+) Caco-2 cells and the downregulation in Annexin A2 (KD) Caco-2 cells were confirmed in protein level in the Caco-2 cells (Figures 2(b) and 2(c)). Then we investigated the influence of the Anxa 2 manipulation (up- or downregulation) on the STAT3 expression. Results demonstrated that the Anxa 2 manipulation exerted no marked regulation in the STAT3 expression in both mRNA and protein levels (*p* < 0.01 or *p* < 0.001, Figures 2(a)–2(c)). However, the phosphorylated STAT3 was markedly upregulated by the Anxa 2 upregulation, whereas it was significantly downregulated by the Anxa 2 downregulation in Caco-2 cells (*p* < 0.01, resp., Figure 2(d)). To investigate the interaction between endogenous phosphorylated Anxa 2 (Tyr 23) and endogenous phosphorylated STAT3 (Tyr 705) in Caco-2 cells with or without Anxa 2 overexpression, coimmunoprecipitation was performed with anti-p-Annexin A2 (Tyr 23) or with anti-p-STAT3 (Tyr 705) in Annexin A2 (+) or control (+) Caco-2 cells. As indicated in Figure 2(e), coimmunoprecipitation results confirmed the p-Annexin A2 (Tyr 23)-p-STAT3 (Tyr 705) interaction in Annexin A2 (+) or control (+) Caco-2 cells. These findings confirmed that phosphorylated Anxa 2 bound to phosphorylated STAT3 in Caco-2 cells. And Anxa 2 was involved in the STAT3 activation in the Caco-2 cells.

### 3.3. STAT3 and Anxa 2 Promote the Proliferation of CRC Caco-2 Cells

**Figure 3:**
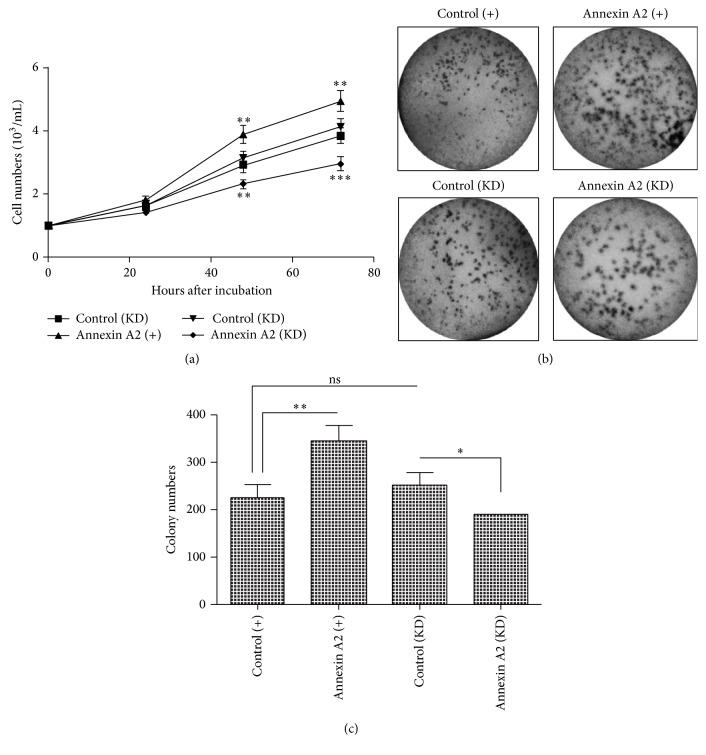
Positive regulation of Annexin A2 in the proliferation of Caco-2 cells. (a) Growth curve of Annexin A2 overexpressed (Annexin A2 (+)), Annexin A2-knocked down (Annexin A2 (KD)), and the two groups of control Caco-2 cells (control (+) and control (KD)), and the cell number was quantified in each group after an incubation of 24, 48, or 72 hours; (b) colony forming assay of the four groups (Annexin A2 (+), control (+), Annexin A2 (KD), and control (KD)) of Caco-2 cells after 48-hour incubation; (c) colony counting of the four groups of Caco-2 cells after 48-hour incubation. Results were repeated in triplicate independently, *∗* represented *p* < 0.05, *∗∗* represented *p* < 0.01, and *∗∗∗* represented *p* < 0.001.

To clarify the regulation by Anxa 2 and STAT3 on tumor growth of CRC, we examined the proliferation of Caco-2 cells, in which Anxa 2 was upregulated or was knocked down, in which STAT3 was knocked down or not. Firstly, the growth curve examination results demonstrated that Caco-2 cells grew more efficiently after the Anxa 2 overexpression (*p* < 0.01, resp.), whereas less efficiently post the Anxa 2 knockdown at both 48 and 72 hours post incubation (*p* < 0.01 or *p* < 0.001, Figure 3(a)). We also detected the differences in colony formation of the Caco-2 cells, after the Anxa 2 overexpression or knockdown. It was shown in Figures 3(b) and 3(c) that more colony numbers were observed in the Annexin A2 (+) group than in the control (+) group (*p* < 0.01), whereas less colonies were formed by the Annexin A2 (KD) Caco-2 cells than the control (KD) Caco-2 cells (*p* < 0.05).

**Figure 4:**
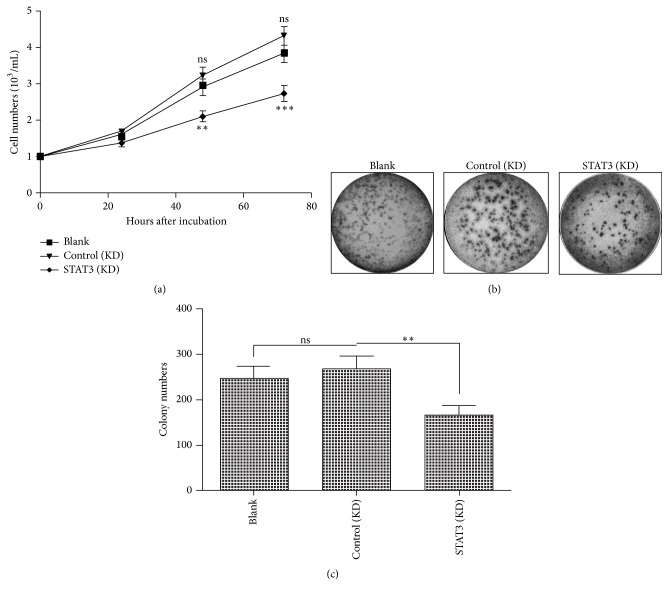
Positive regulation of STAT3 in the proliferation of Caco-2 cells. (a) Growth curve of blank Caco-2 cells (as blank control), Caco-2 cells which were transfected with sh-STAT3 (STAT3 (KD)), or control shRNA (control (KD)), and the cell number was counted in each group after an incubation of 24, 48, or 72 hours; (b) colony forming assay of blank, STAT3 (KD), and control (KD) Caco-2 cells after 48-hour incubation; (c) colony counting of the three groups of Caco-2 cells. Results were repeated in triplicate independently, *∗∗* represented *p* < 0.01, *∗∗∗* represented *p* < 0.001, and ns represented no significance.

Secondly, we instigated the regulation of the STAT3 knockdown on the proliferation of Caco-2 cells. The growth curve results indicated that there was no significant difference in the proliferation efficiency between the blank and control shRNA-transfected (control (KD)) Caco-2 cells. However, the Caco-2 cells which were transfected with shRNA-STAT3 (STAT3 (KD)) proliferated less efficiently than the control (KD) Caco-2 cells (*p* < 0.01 or *p* < 0.001, Figure 4(a)). And the colony forming assay reconfirmed such difference in the proliferation of Caco-2 cells. Neither significant difference in the colony formed by Caco-2 cells was observed between the blank and control (KD) groups (Figures 4(b) and 4(c)). However, the STAT3 (KD) Caco-2 cells formed fewer colonies than the control (KD) Caco-2 cells (*p* < 0.01, Figures 4(b) and 4(c)). Therefore, both Anxa 2 and STAT3 promote the proliferation of CRC Caco-2 cells.

### 3.4. STAT3 and Anxa 2 Regulated the Migration and Invasion of CRC Caco-2 Cells

**Figure 5:**
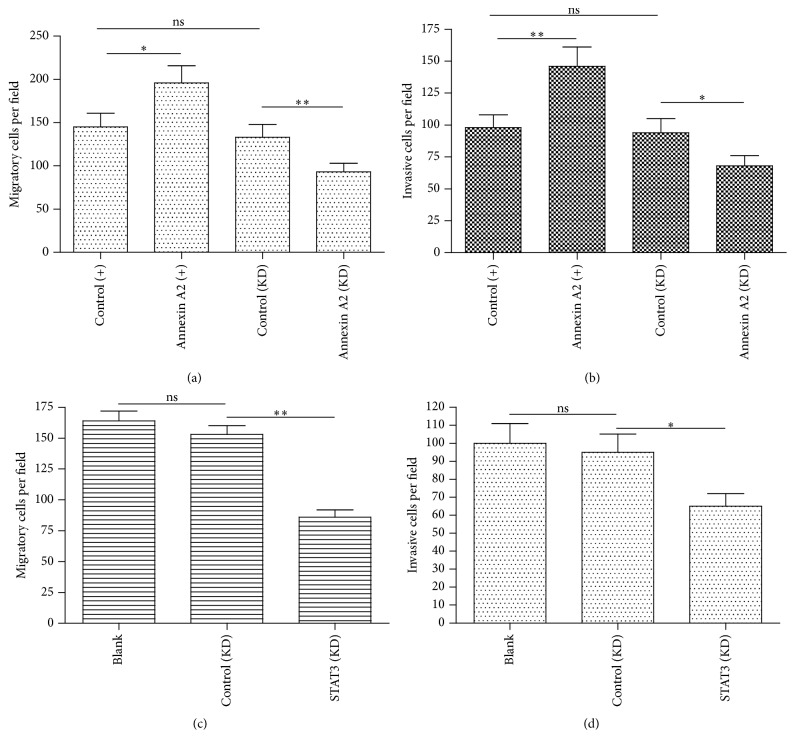
Annexin A2 and STAT3 regulate the migration and invasion of CRC Caco-2 cells. The number of Caco-2 cells was calculated to compare the differences in migration and invasion of Caco-2 cells with indicated treatments by using transwell migration/invasion assays. The effects of Annexin A2 on cells migration (a) and invasion (b) and the effects of STAT3 on cells migration (c) and invasion (d) were indicated, respectively. The three individual experiments were performed. Statistical significance was shown as ^*∗*^ *p* < 0.05 and ^*∗∗*^ *p* < 0.01.

To investigate the effect of STAT3 and Anxa 2 on tumor metastasis, we observed the migration and invasion of CRC Caco-2 cells, after the manipulation of STAT3 and Anxa 2 by transwell migration/invasion assays. The difference in the migration among CRC cells with Anxa 2 knockdown and Anxa 2 overexpression and without treatment was observed. Upregulated Anxa 2 promoted the Caco-2 cells migration significantly (*p* < 0.05 for Annexin A2 (+) versus control (+), Figure 5(a)). Moreover, there was dramatically lower migration in Annexin A2 knocked down Caco-2 cells than in the control Caco-2 (*p* < 0.01 for Annexin A2 (KD) versus control (KD), Figure 5(a)). In addition, the effect of Anxa 2 on the invasion of Caco-2 cells was consistent with that on migration as shown in Figure 5(b) (*p* < 0.01 for Annexin A2 (+) versus control (+), *p* < 0.05 for Annexin A2 (KD) versus control (KD)). The data demonstrated that upregulated Anxa 2 could promote metastasis and inhibiting Anxa 2 could repress the metastasis of colon cancer cells. In addition, we examined the migration and invasion of Caco-2 cells in which the STAT3 was knocked down or not. Figures 5(c) and 5(d) demonstrated that there were no significant differences in the migratory and invasive cell counting between the blank and control (KD) Caco-2 cells; however, the STAT3 (KD) markedly reduced both migratory and invasive cells (*p* < 0.01 or *p* < 0.05). Owing to above results on the highly positive association of Anxa 2 and STAT3 with the invasion and migration of Caco-2 cells, we concluded that Anxa 2 coordinated STAT3 to regulate the invasion and migration in colon cancer cells.

## 4. Discussion

Dysregulation of Annexin A2 [21, 22] and STAT3 [29, 30] has been reported to take effects on cancer progression, metastasis, and prognosis, particularly in CRC. STAT3 takes intrinsic activator effects on cancer inflammation and regulates the tumor microenvironment [7, 8]. In this study, we found the upregulation of both Annexin A2 and STAT3 with or without phosphorylation in CRC tissues, compared to the paratumor tissues. These data confirmed the overexpression of Annexin A2 and STAT3 in CRC* in vivo*. It also meant these molecules might exert important role in the tumorigenesis of CRC.

Activated STAT3 leads to dramatically increased expression of STAT3 and its downstream genes which generate biological effects and feedback to stimulate the expression of STAT3 [31], linking inflammation to epithelial cancer. Thus, the initiator of the STAT3 activation might be a key regulator of the STAT3-mediated tumorigenesis. In the present study, we found that, along with the overexpression of STAT3, Annexin A2 was also overexpressed in the CRC specimens. And the active form of both markers, the phosphorylated Annexin A2 (Tyr 23) and the phosphorylated STAT3 (Tyr 705), was also upregulated in CRC tissues. Moreover, the* in vitro* experiments confirmed the promotion by the Annexin A2 overexpression on the STAT3 phosphorylation (Tyr 705) in CRC Caco-2 cells. And the coimmunoprecipitation confirmed the binding of phosphorylated Anxa 2 to phosphorylated STAT3 in Caco-2 cells. Thus, we speculated Annexin A2 as a key initiator of STAT3 signaling in the tumorigenesis of CRC.

Proliferation, invasion, and metastasis lead to the growth and dissemination of cancer cells. The important oncogenic role of Annexin A2 has been recognized in various types of tumors [11, 17, 19, 21]. In the present study, the combined strategies of gain-of-function and loss-of-function confirmed the promotion by Annexin A2 on the proliferation, invasion, and migration of CRC cells. The overexpressed Annexin A2 markedly promoted and the knockdown of it reduced the proliferation, invasion, and migration of Caco-2 cells* in vitro*. STAT3 is constitutively activated in many cancers and plays a pivotal role in tumor growth and metastasis [32]. In the present study, we also confirmed the promotion by STAT3 on the proliferation, invasion, and migration of CRC Caco-2 cells via the loss-of-function strategy.

In conclusion, Annexin A2 coordinated with STAT3 signaling to regulate the proliferation, invasion, and migration of CRC cells. It firstly revealed the coordination of Annexin A2 and STAT3 in CRC cells, and such two coordinated molecules modulate the proliferation, invasion, and metastasis in CRC.

## Figures and Tables

**Table 1:** Clinicopathologic information of colorectal cancer patients.

Characteristics	CRC (*n* = 35)
Gender	
Male	19
Female	16
Age (range, years)	46 (34–61)
Tumor stage^*∗*^	
I	4
IIA	16
IIIA	6
IIIB	8
IIIC	1
Tumor grade	
G1	4
G2	24
G3	7

^*∗*^NCCN stage: stage I:  T_1_-T_2_N_0_M_0_; stage IIA: T_3_N_0_M_0_; stage IIIA: T_1_-T_2_N_1/1c_M_0_ or T_1_N_2a_M_0_; stage IIIB: T_3-4a_N_1/1c_M_0_, T_2_-T_3_N_2a_M_0_, or T_1_-T_2_N_2b_M_0_; stage IIIC: T_4a_N_2a_M_0_, T_3_-T_4a_N_2b_M_0_, or T_4b_N_1_-T_2_M_0_.
